# Association between disease activity of rheumatoid arthritis and maternal and fetal outcomes in pregnant women: a systematic review and meta-analysis

**DOI:** 10.1186/s12884-023-06033-2

**Published:** 2023-10-11

**Authors:** Jiamin Lv, Li Xu, Shuhui Mao

**Affiliations:** 1 Department of Obstetrics, Jiaxing Women and Children’s Hospital of Jiaxing University, No.2468 Middle Ring East Road, Nanhu District, Jiaxing, 314051 P.R. China; 2 Department of Internal Medicine, Jiaxing Women and Children’s Hospital of Jiaxing University, Jiaxing, 314051 P.R. China

**Keywords:** Rheumatoid arthritis, High disease activity, Maternal outcomes, Fetal outcomes, meta-analysis

## Abstract

**Background:**

A meta-analysis has compared the pregnancy outcomes between women with and without RA, while the effect of disease severity on pregnancy outcomes within women with RA has not been explored. Therefore, we performed a systematic review and meta-analysis to assess the association between disease activity of RA and pregnancy outcomes.

**Methods:**

Four English databases (Pubmed, Embase, Cochrane Library, and Web of Science) and three Chinese databases (China National Knowledge Infrastructure [CNKI], VIP, and Wan Fang) was searched for eligible studies up to August 13, 2023. Cochran’s Q test and the I^2^ statistic were used to assess the heterogeneity of the included studies. The odds ratio (OR) (for counting data) and weighted mean difference (WMD) (for measurement data) were calculated with 95% confidence intervals (95%CIs) using random-effect model (I^2^ ≥ 50%) or fixed-effect model (I^2^ < 50%). Subgroup analysis based on study design and regions was used to explore the sources of heterogeneity. Sensitivity analysis was performed for all outcomes and the publication bias was assessed using Begg’s test.

**Results:**

A total of 41 eligible articles were finally included. RA women had higher odds to suffer from preeclampsia, gestational diabetes, spontaneous abortion, and cesarean delivery (all *P* < 0.05). The infants born from RA mother showed the higher risk of stillbirth, SGA, LBW, congenital abnormalities, diabetes type 1, and asthma (all *P* < 0.05). The high disease activity of RA was significantly associated with the higher risk of cesarean delivery (OR: 2.29, 95%CI: 1.02–5.15) and premature delivery (OR: 5.61, 95%CI: 2.20–14.30).

**Conclusions:**

High disease activity of RA was associated with the high risk of adverse pregnancy outcomes, suggesting that it was important to control disease for RA women with high disease activity who prepared for pregnancy.

## Background

Rheumatoid arthritis (RA) is a chronic autoimmune disease, which is characterized by synovial inflammation, cartilage damage, and bone erosion, and leads to severe physical disability [1]. The estimated prevalence of RA is 0.5-1.0% worldwide, and women are twice as likely to suffer from RA than men, with most cases occurring in women of childbearing age [1]. RA impairs the fertility, and compared to the general population, pregnancy outcomes are not satisfactory in women with RA, especially in those with high disease activity [2].

Several studies have reported the correlation between RA and adverse pregnancy outcomes [3–6]. A meta-analysis has reported that maternal RA increased the risk of autism spectrum disorders in offspring [7]. However, this meta-analysis has not reported the maternal outcomes and other fetal outcomes [7]. A meta-analysis performed by Huang et al. showed that maternal RA was significantly correlated with an increased risk of adverse maternal and fetal outcomes [8]; however, the association between disease activity and pregnancy outcomes was not explored in their meta-analysis.

Existing studies have shown that higher disease activity of RA was correlated with the higher risk of adverse pregnancy outcomes [9, 10]. A study by de Man et al. has reported that pregnancy outcomes of women with well-controlled RA was comparable with those of the general population [11]. A study by Langen et al. showed no association between disease activity and pregnancy outcomes in RA women, but they found that medication discontinuation increased the odds of adverse pregnancy outcomes at delivery [12]. Previous meta-analyses have reported pregnancy outcomes of women with and without RA without considering the disease severity [7, 8]. Given that it is important to assess pregnancy outcomes by disease status, there is a need to further examine the effect of disease severity on pregnancy outcomes within a population of women with RA.

We performed a systematic review and meta-analysis based on current available publications to systematically assess the association between the disease activity of RA and pregnancy outcomes in women with RA. We also examined the pregnancy outcomes in women with and without RA. The combination and analysis of data on this issue may provide useful clinical management and counselling for RA women.

## Methods

The standard Cochrane methods were used in this meta-analysis, which performed according to Preferred Reporting Items for Systemic Reviews and Meta-Analyses (PRISMA) guideline [13]. The protocol of this meta-analysis was registered at PROSPERO (registration number: CRD42023402272).

### Literature search strategy

Two researchers (JML and LX) searched the studies in four English databases (PubMed, Embase, Web of Science, and Cochrane Library) and three Chinese databases (China National Knowledge Infrastructure [CNKI], VIP, and Wan Fang) from inception to August 13, 2023. The search strategy included: “Arthritis, Rheumatoid” OR “Rheumatoid Arthritis” AND “Pregnancy” OR “Pregnancies” OR “Gestation” OR “Pregnancy Outcome” OR “Pregnancy Outcomes” OR “Outcome, Pregnancy” OR “Outcomes, Pregnancy” OR “Maternal outcomes” OR “Fetal outcomes”.

### Inclusion and exclusion criteria

Studies were included if they met all the following criteria: (1) population: pregnant women with and without RA; (2) exposure and comparator: women with RA vs. women without RA, RA women with high disease activity vs. RA women with low disease activity; (3) outcome: adverse maternal and/or fetal outcomes; (4) study: observational studies; (5) language: published in English or Chinese.

Disease activity was assessed using Disease Activity Score-28 (DAS28), Health Assessment Questionnaire-Disability Index (HAQ-DI), pain score (PS), and patient’s global scale (PGS). DAS28 > 3.2 and HAQ-DI > 0.5 were defined as high disease activity [14].

Maternal outcomes included preeclampsia, gestational diabetes, hypertension, spontaneous abortion (pregnancy loss before 28 weeks of gestation), cesarean delivery, postpartum infection, postpartum hemorrhage, and maternal depression. Fetal outcomes included premature delivery (delivery at 28–37 weeks of gestation), stillbirth (delivery of a dead fetus at > 27 weeks of gestation), neonatal death within 30 days of birth, small for gestational age (SGA), birth weight, LBW (birth weight < 2500 g), low Apgar score (score at 5 min < 7), requiring intensive care, infantile autism (IA), congenital abnormalities, RA, Juvenile idiopathic arthritis (JIA), diabetes type 1, asthma, and epilepsy.

Studies were excluded if they met one of the following criteria: (1) animal studies; (2) topic not meeting the requirements; (3) with incomplete data (data categories not meeting our requirements) or unable to extract data (contacting the authors for many times but no reply to obtain the original text); (4) duplicates of the same studies; (5) conferences, abstract, case reports, meta-analysis, and review.

### Data extraction

Two of the authors (JML and LX) independently evaluated the data reported in the publications which were suitable for this research and cross-checked to ensure that no data were missed. The following data were extracted: the first author, publication year, region that the study performed, study design, total number of participants, separate number of women with RA and without RA, maternal age, maternal outcomes, and fetal outcomes. The third author (SHM) participated and resolved the disagreements by consensus.

### Methodological quality appraisal

The quality of cohort studies and case-control studies was assessed using the Newcastle-Ottawa Scale (NOS), which was a nine-point scale and divided studies into poor quality (0–3 points), fair quality (4–6 points), and good quality (7–9 points) [15]. The quality of cross-sectional studies was evaluated using the Joanna Briggs Institute (JBI), which was a 20-point scale and divided studies into low quality (0–14 points) and high quality (15–20 points) [16].

### Statistical analysis

The comparison results of categorical data were expressed as odds ratio (OR) and 95% confidence intervals (95%CIs), and the comparison results of continuous data were expressed as weighted mean difference (WMD) with 95%CIs. Cochran’s Q test and the I^2^ statistic were used to assess the heterogeneity between the studies. Random-effect model was used if heterogeneity was found (I^2^ values ≥ 50%) and fixed-effect model was used if I^2^ values < 50%. Subgroup analysis based on study design and regions was performed to identify the sources of heterogeneity. Sensitivity analysis was performed for all outcomes and publication bias was assessed using Begg’s test if more than nine studies were included [17]. Data were analyzed using STATA v15.1 (STATA Corporation, College Station, TX, USA). *P* < 0.05 was considered as statistical significance.

## Results

### Literature search and study characteristics

**Fig. 1:**
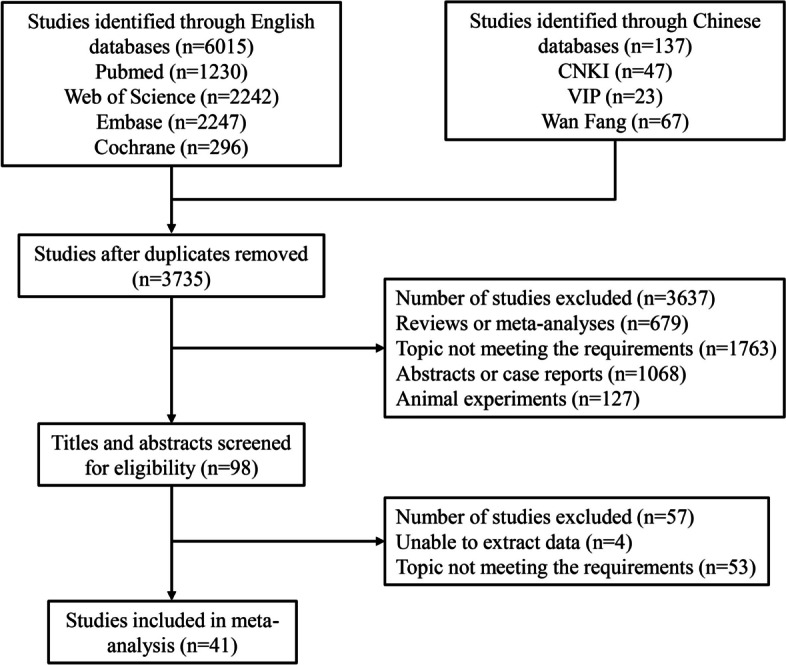
The flowchart of the studies selection

From the above-mentioned four English databases and three Chinese databases, 6,015 English articles and 137 Chinese articles were obtained. After removing the duplicates, 3,735 articles remained. By screening titles and abstracts, 3,637 articles were excluded because they were reviews or meta-analyses (*n* = 679), their topic did not meet the requirements (*n* = 1763), abstracts or case reports (*n* = 1068), or animal experiments (*n* = 127). Further, 57 articles were eliminated due to data unable to extract (*n* = 4) and topic not meeting the requirements after a careful assessment of full texts (*n* = 53). Finally, 41 eligible articles were included in the meta-analysis (Fig. 1) [3–6, 9–11, 14, 18–50]. Of the included articles, there were 32 cohort studies, 7 case-control studies, and 2 cross-sectional studies. For quality assessment of the studies, 14 studies, 25 studies, and 2 studies were assessed as good, fair, and poor quality, respectively (Table 1).

**Table 1:** The characteristics of the included studies

Author	Year	Region	Study design	Total	Women with RA		Women without RA		Maternal outcomes	Fetal outcomes	Quality assessment
					N	Maternal age	N	Maternal age			
Bowden	2001	UK	case-control	236	133	32.7 ± 4.6	103	30.0 ± 4.5		birth weight	5
Reed	2006	USA	cohort	2802	243	NA	2559	NA	cesarean delivery, preeclampsia	premature, LBW, SGA infant	4
Mouridsen	2007	Denmark	case-control	441	7	NA	434	NA		infantile autism	7
de Man	2009	Netherlands	cohort	3811	152	32.5 ± 3.7	3659	31.2 ± 4.5		birth weight	6
Atladóttir	2009	Denmark	cohort	689,196 children	NA	NA	NA	NA		autism spectrum disorder	5
Lin	2010	China	cohort	11,472	1912	NA	9560	NA	cesarean delivery, preeclampsia	premature, LBW, SGA infant	8
Nørgaard	2010	Sweden, Denmark	cohort	871,579	1199	NA	870,380	NA	cesarean delivery, preeclampsia, gestational diabetes	premature, Apgar score at 5 min below 7, SGA infant, stillbirth, congenital abnormalities	4
Barnabe	2011	Canada	cohort	188	38	32 ± 5.5	150	NA	cesarean delivery, preeclampsia, gestational diabetes, postpartum infection	premature, requiring intensive care, SGA infant, congenital defects	5
Ma	2014	USA	cohort	1304	202	NA	1102	NA		premature, LBW, SGA infant	7
Bharti	2015	USA	cohort	440	440	32.7 ± 4.6			cesarean delivery	premature, SGA infant	5
Pósfai	2015	Hungary	case-control	38,151	68	26.9 ± 5.6	38,083	25.5 ± 5.3	gestational diabetes, hypertension	birth weight, preterm birth, LBW, congenital abnormalities	3
Wallenius	2015	Norway	cohort	412,708	1578	32.1 ± 4.8	411,130	30.9 ± 5.1	spontaneous abortion	stillbirth	5
Atta	2015	Egypt	cohort	69	47	31.1 ± 1.4	22	32.7 ± 0.9	cesarean delivery	premature, SGA	4
Rom-a	2016	Denmark	cohort	1,917,723	13,556	28.46 ± 5.1	1,904,167	28.35 ± 4.9		Juvenile Idiopathic Arthritis, Diabetes type 1, Asthma	8
Rom-b	2016	Denmark	cohort	1,909,933	13,511	28 ± 5.1	1,896,422	28 ± 4.9		Apgar score below 7, epilepsy	7
Tsai	2017	China	cohort	1,893,244	673	31.97 ± 4.51	1,892,571	29.50 ± 4.80		autism spectrum disorder	6
Bandoli	2017	USA	cohort	2432	729	32.5 ± 4.8	1703	32.1 ± 5.1	preeclampsia, maternal depression, gestational diabetes	premature, birth weight	5
Galappatthy	2017	Sri Lanka	cohort	165	80	35 ± 6.7	85	NA	spontaneous abortion, hypertension, gestational diabetes	stillbirth, LBW	5
Eudy	2017	USA	cross-sectional	150	75	32.0 ± 5.2	75	31.9 ± 5.1	spontaneous abortion, gestational diabetes, cesarean delivery, preeclampsia	premature, congenital abnormalities, NICU visit	15
Jølving	2018	Denmark	cohort	1,380,645	2106	NA	1,378,539	NA	cesarean delivery	premature, SGA infant, rheumatoid arthritis, diabetes mellitus, epilepsy	7
Rom	2018	Denmark	cohort	1,917,723	13,556	28.46 ± 5.1	1,904,167	28.35 ± 4.9		autism spectrum disorder	8
Lin	2018	China	cohort	2707	34	NA	2673	NA	postpartum depression		5
Zbinden	2018	Switzerland	cohort	156	86	32 (22–44)	70	32 (20–41)	cesarean delivery	premature, SGA infant	7
Smith	2018	USA, Canada	cohort	1221	657	33.14 ± 4.67	564	32.09 ± 4.7	cesarean delivery, preeclampsia, gestational diabetes, hypertension	premature	5
Aljary	2018	Canada	cohort	847,607	6068	NA	841,539	NA	cesarean delivery, preeclampsia, gestational diabetes, hypertension, postpartum hemorrhage	premature, SGA infant	5
Croen	2019	USA	case-control	1578	15	NA	1563	NA		autism spectrum disorder	7
Strouse	2019	USA	cohort	13,165	2921	NA	10,244	NA		premature, congenital anomalies, LBW, SGA infant	6
Keeling	2019	Canada	cohort	309,620	631	30.4 ± 5.6	308,989	29.3 ± 8.4	cesarean delivery, gestational diabetes, hypertension	premature, SGA infant, birth weight, neonatal death within 30 days of birth, congenital anomaly	5
Knudsen	2019	Denmark	cohort	690,240	1026	NA	689,214	NA		premature, birth weight	6
Nathan	2019	Denmark	cohort	2,584,932	3749	NA	2,581,183	NA	spontaneous abortion		6
Abdulrahman	2020	Egypt	cross-sectional	300	200	37.84 ± 6.67	100	NA	preeclampsia, gestational diabetes, cesarean delivery	LBW, paediatric ICU admission, congenital anomalies	11
Bortoluzzi	2020	Italy	cohort	6540	443	34 (31–37)	6097	34 (30–37)		miscarriage and perinatal death	5
Knudsen	2020	Denmark	cohort	738,862	934	30.01 ± 4.78	737,928	31.48 ± 4.67		birth weight, LBW, premature, congenital abnormalities	6
Al Rayes	2021	Saudi Arabia	cohort	327	77	32.78 ± 0.69	250	30.32 ± 0.84	preeclampsia, spontaneous abortion, cesarean delivery	newborn weight, congenital abnormalities, stillbirth, preterm birth, NICU admission	7
Yang	2021	China	case-control	628,878	1188	29.1 ± 4.59	627,690	29.1 ± 4.59		asthma	5
Park	2022	Korea	cohort	27,675	1652	32.3 ± 3.8	26,023	31.8 ± 4.0	preeclampsia, spontaneous abortion, cesarean delivery	premature, LBW	6
Tarplin	2022	USA	cohort	798	202	31 ± 5	596	27 ± 7	preeclampsia, spontaneous abortion, cesarean delivery	premature, stillbirth	5
Tsai	2022	China	cohort	2,100,143	922	32.43 ± 4.42	2,099,221	30.18 ± 4.75	preeclampsia, hypertension	stillbirth, LBW, premature, SGA infant, fetal abnormalities	5
Singh	2023	USA	cohort	13,516	1223	NA	12,293	NA	preeclampsia, gestational diabetes, cesarean delivery	premature, LBW, SGA infant, Apgar score below 7, fetal abnormalities	7
Raitio	2023	Finland	case-control	1140	8	NA	1132	NA		congenital anomalies	6
Bobircă	2023	Romania	case-control	365	66	31.3 ± 4.4	299	29.2 ± 5.5	cesarean delivery	premature, SGA, LBW	7

*RA *Rheumatoid arthritis, *LBW *Low birth weight, *SGA *Small for gestational age, *NICU *Neonatal intensive care unit, *ICU *Intensive care unit, *NA *Not available

### Systematic review and meta-analysis of the association between maternal RA and adverse maternal/fetal outcomes

Table 2 shows that RA was associated with an increased risk of preeclampsia (OR: 1.65, 95%CI: 1.53–1.78, I^2^ = 13.4%), gestational diabetes (OR: 1.61, 95%CI: 1.25–2.07, I^2^ = 72.0%), spontaneous abortion (OR: 1.32, 95% CI: 1.21–1.43, I^2^ = 25.8%), and cesarean delivery (OR: 1.62, 95%CI: 1.43–1.84, I^2^ = 87.9%). Study design or region was the source of heterogeneity for gestational diabetes and cesarean delivery. Table 3 displays that RA was associated with an increased risk of stillbirth (OR: 1.55, 95% CI: 1.17–2.06, I^2^ = 0.0%), SGA (OR: 1.48, 95%CI: 1.25–1.75, I^2^ = 85.4%), LBW (OR: 1.73, 95% CI: 1.46–2.06, I^2^ = 65.8%), congenital abnormalities (OR: 1.24, 95% CI: 1.13–1.37, I^2^ = 42.3%), diabetes type I (OR: 1.70, 95% CI: 1.41–2.06, I^2^ = 36.8%), and asthma (OR: 1.23, 95% CI: 1.16–1.30, I^2^ = 0%). Study design or region was the source of heterogeneity for birth weight and LBW.

**Table 2:** Summary results of the association between rheumatoid arthritis and maternal outcomes

Outcomes	Number of studies	OR (95%CI)	*P*	I^2^ (%)
Preeclampsia [9, 10, 18, 20, 21, 26, 30, 35, 36, 43–46, 49]	14	1.65 (1.53, 1.78)	< 0.001	13.4
Gestational diabetes [4, 10, 18, 20, 26, 27, 35, 46, 49, 50]	10	1.61 (1.25, 2.07)	< 0.001	72.0
Study design				
Cohort study		1.43 (1.17, 1.75)	0.001	63.9
Case-control study		10.52 (3.80, 29.12)	< 0.001	NA
Cross-sectional study		2.04 (0.40, 10.40)	0.393	0.0
Region				
Europe		5.05 (1.38, 18.54)	0.015	80.5
North America		1.35 (1.14, 1.61)	0.001	62.2
Asia		0.53 (0.05, 5.91)	0.602	NA
Africa		2.02 (0.22, 18.32)	0.532	NA
Hypertension [4, 10, 27, 38, 49, 50]	6	0.66 (0.14, 3.14)	0.597	99.2
Spontaneous abortion [9, 26, 27, 40, 43, 44]	6	1.32 (1.21, 1.43)	< 0.001	25.8
Cesarean delivery [6, 9, 10, 18, 21, 26, 30, 35, 36, 42–44, 46, 48–50]	16	1.62 (1.43, 1.84)	< 0.001	87.9
Study design				
Cohort study		1.63 (1.43, 1.86)	< 0.001	89.8
Cross-sectional study		1.34 (0.36, 4.93)	0.663	85.2
Case-control study		1.44 (0.84, 2.46)	0.183	NA
Region				
Europe		1.97 (1.63, 2.38)	< 0.001	68.5
North America		1.60 (1.42, 1.80)	< 0.001	61.6
Asia		1.23 (1.14, 1.32)	< 0.001	0.0
Africa		2.51 (1.44, 4.38)	0.001	NA
Maternal depression [14, 20]	2	1.64 (0.84, 3.20)	0.146	69.6

*OR* Odds ratio, *CI* Confidence intervals, *I*^2^ I-squared, *NA* Not available

**Table 3:** Summary results of the association between rheumatoid arthritis and fetal outcomes

Outcomes	Number of studies	OR/WMD (95%CI)	*P*	I^2^ (%)
Premature delivery [3, 4, 6, 9, 10, 20, 21, 26, 28–30, 32, 35, 36, 42–46, 48–50]	22	1.57 (1.00, 2.48)	0.052	98.8
Stillbirth [9, 27, 35, 38, 40, 44]	6	1.55 (1.17, 2.06)	0.003	0.0
SGA [3, 6, 21, 30, 32, 35, 36, 42, 45, 46, 48–50]	13	1.48 (1.25, 1.75)	< 0.001	85.4
Region				
Europe		1.55 (1.15, 2.09)	0.004	53.9
North America		1.46 (1.12, 1.89)	0.005	89.5
Asia		1.42 (1.01, 2.00)	0.047	89.7
Birth weight [4, 9, 20, 24, 28, 29, 50]	7	-135.10 (-244.27, -25.94)	0.015	97.7
Study design				
Cohort study		-173.78 (-295.12, -52.43)	0.005	98.2
Case-control study		-23.25 (-330.96, 284.47)	0.882	92.6
Region				
Europe		-71.64 (-146.99, 3.72)	0.062	82.9
North America		-162.00 (-235.49, -88.51)	< 0.001	78.6
Asia		-342.59 (-362.97, -322.22)	< 0.001	NA
LBW [3, 4, 18, 27, 29, 30, 32, 36, 43, 45, 46, 48]	12	1.73 (1.46, 2.06)	< 0.001	65.8
Study design				
Cohort study		1.75 (1.46, 2.09)	< 0.001	72.2
Case-control study		1.16 (0.29, 4.57)	0.833	66.4
Cross-sectional study		1.99 (0.72, 5.51)	0.183	NA
Region				
Europe		1.41 (0.87, 2.28)	0.161	33.0
North America		1.80 (1.45, 2.25)	< 0.001	52.8
Asia		1.72 (1.19, 2.49)	0.004	84.8
Africa		1.99 (0.72, 5.51)	0.183	NA
Apgar score at 5 min below 7 [35, 38, 46]	3	1.05 (0.71, 1.54)	0.818	63.2
Requiring intensive care [9, 18, 21, 26]	4	1.93 (0.82, 4.56)	0.133	56.0
Infantile autism [25, 33, 39]	3	1.41 (0.76, 2.61)	0.278	0.0
Congenital abnormalities [3, 4, 9, 18, 21, 26, 29, 35, 45–50]	12	1.24 (1.13, 1.37)	< 0.001	42.3
Diabetes type 1 [5, 6]	2	1.70 (1.41, 2.06)	< 0.001	36.8
Asthma [5, 41]	2	1.23 (1.16, 1.30)	< 0.001	0.0

*OR *Odds ratio, *CI *Confidence intervals, *WMD *Weighted mean difference, *I*^2 ^I-squared, *SGA *Small for gestational age, *LBW *Low birth weight, *NA *Not available

Barnabe et al. reported no statistical difference in the risk of postpartum infection between pregnant women with RA and without RA (*P* = 0.875) [21]. Aljary et al. found no significant difference in the risk of postpartum hemorrhage between women with RA and without RA (*P* = 0.276) [49]. In addition, there was no significance in the risk of neonatal death within 30 days of birth (*P* = 0.477) [50] and epilepsy (*P* = 0.164) [6], while risks of RA [6] and JIA [5] were higher in infants born from mother with RA.

### Systematic review and meta-analysis of the association between disease activity of RA and maternal/fetal outcomes

**Fig. 2:**
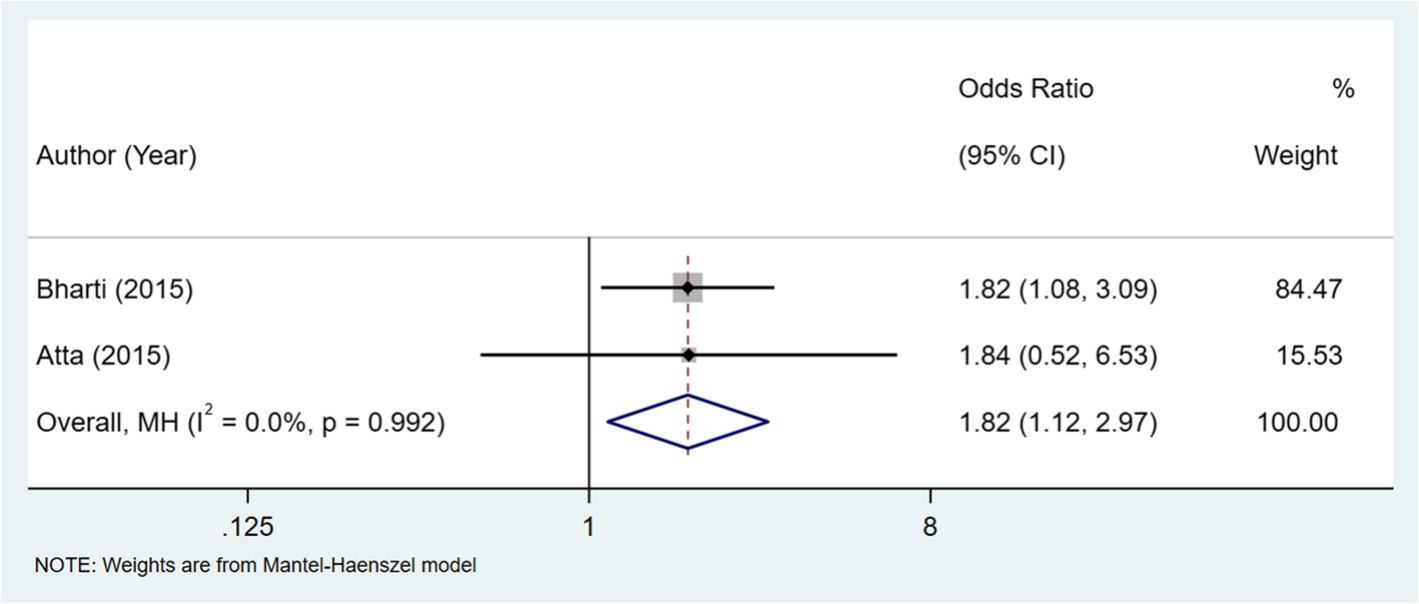
Forest plot for the association between the disease activity of RA assessed by HAQ-DI and premature delivery

Table 4 summarizes the pooled results on the association between disease activity of RA and maternal or fetal outcomes. HAQ-DI > 0.5 was associated with an increased risk of premature delivery (OR: 1.82, 95% CI: 1.12–2.97, I^2^ = 0%), indicating that high disease activity of RA was significantly associated with the high risk of premature delivery. The forest plot regarding premature delivery by HAQ-DI was shown in Fig. 2.

**Table 4:** Summary results of the association between disease activity of rheumatoid arthritis and maternal/fetal outcomes

Outcomes	Number of studies	OR (95%CI)	*P*	I^2^ (%)
HAQ-DI (> 0.5 vs. ≤ 0.5)				
Cesarean delivery [14, 22]	2	1.34 (0.92, 1.96)	0.131	0.0
Premature delivery [14, 22]	2	1.82 (1.12, 2.97)	0.016	0.0
SGA infant [14, 22]	2	3.06 (0.88, 10.66)	0.078	55.0
DAS28 (> 3.2 vs. ≤ 3.2)				
Cesarean delivery [14, 42]	2	2.29 (1.02, 5.15)	0.044	0.0
Premature delivery [14, 42]	2	5.61 (2.20, 14.30)	< 0.001	17.8
SGA infant [14, 42]	2	6.36 (0.18, 226.24)	0.310	81.3

*HAQ-DI *Health Assessment Questionnaire-Disability Index, *DAS28 *Disease Activity Score-28, *OR *Odds ratio, *CI *Confidence intervals, *I*^2 ^ I-squared, *SGA *Small for gestational age

**Fig. 3:**
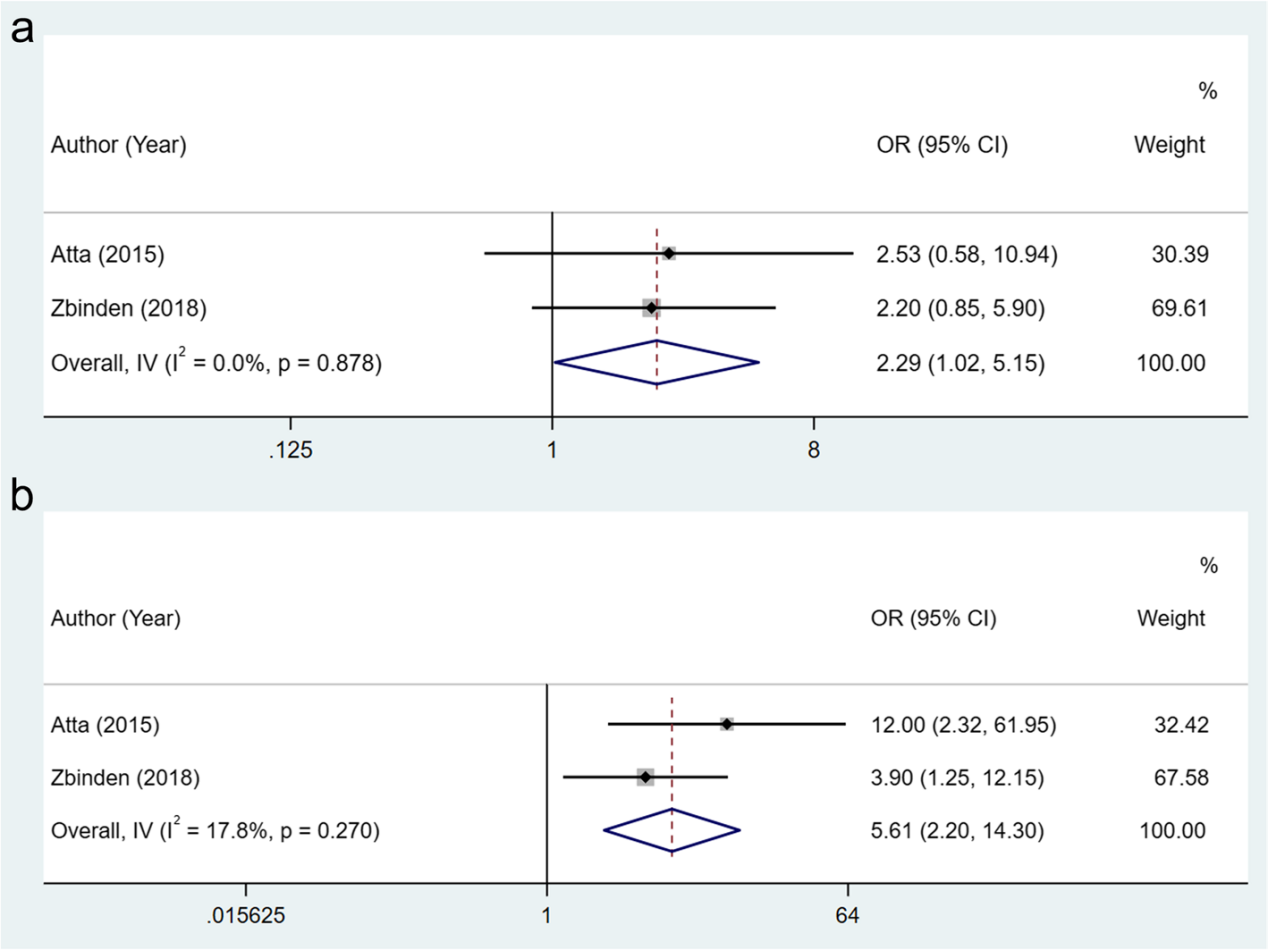
Forest plots for the association between the disease activity of RA assessed by DAS28 and cesarean delivery (**A**) and premature delivery (**B**)

We also found that there was a significant increase in the risk of cesarean delivery (OR: 2.29, 95% CI: 1.02–5.15, I^2^ = 0%) and premature delivery (OR: 5.61, 95% CI: 2.20–14.30, I^2^ = 17.8%) in RA women with DAS28 > 3.2, which reflected the unfavorable effect of high disease activity of RA on the cesarean delivery and premature delivery. Forest plots regarding cesarean delivery and premature delivery by DAS28 were shown in Fig. 3A and B.

De Man et al. reported that higher disease activity of RA showed a relationship with the lower birth weight of newborns [11]. Smith et al. reported that the increase of disease activity was associated with the increased risk of premature delivery [10]. In addition, Al Rayes found that the higher disease activity of RA was associated with the higher risk of spontaneous abortion, premature delivery, and neonatal intensive care unit (NICU) admission [9].

### Sensitivity analysis and publication bias

The sensitivity analysis was carried out by sequentially excluding one of the studies each time, and the results were consistent, indicating every included study had equal sensitivity and did not impact the overall results (data not shown). Begg’s test revealed that there was no publication bias regarding to the risk of preeclampsia (Z = 0.66, *P* = 0.511), gestational diabetes (Z = 0.72, *P* = 0.474), cesarean delivery (Z = 0.05, *P* = 0.964), premature delivery (Z = 1.64, *P* = 0.102), SGA infant (Z = 1.04, *P* = 0.300), LBW (Z = 0.89, *P* = 0.373), and congenital abnormalities (Z = 0.34, *P* = 0.732) (Table 5).

**Table 5:** Publication bias of outcomes by Begg’s test

Outcomes	Begg’s test	
	Z	*P*
Preeclampsia	0.66	0.511
Gestational diabetes	0.72	0.474
Cesarean delivery	0.05	0.964
Premature delivery	1.64	0.102
SGA infant	1.04	0.300
LBW	0.89	0.373
Congenital abnormalities	0.34	0.732

*SGA *Small for gestational age, *LBW *Low birth weight

## Discussion

In this systematic review and meta-analysis, we systematically assessed the association between RA and pregnancy outcomes, and we also quantified the data on the association between the disease activity of RA and pregnancy outcomes. The results showed that pregnant women with RA had a higher risk of preeclampsia, gestational diabetes, spontaneous abortion, and cesarean delivery than those without RA. Infants born from RA mother had a higher risk of stillbirth, SGA, LBW, congenital abnormalities, type 1 diabetes, and asthma than those born from mother without RA. In addition, we found that high disease activity of RA was associated with the higher risk of premature delivery and cesarean delivery.

Studies have reported the more prevalence of type 2 diabetes mellitus in RA patients than non-RA affected people [49, 51]. In our analysis, we found a higher risk of gestational diabetes in women with RA. This may be because that disease activity in pregnant RA women was usually controlled using glucocorticoids, which may decrease the sensitivity of peripheral insulin, increase the production of hepatic glucose, and inhibit the production and secretion of pancreatic insulin [52], thereby promoting the development of diabetes in patients with RA [53, 54]. Moreover, preeclampsia was commonly observed in RA women. The reason for the association between preeclampsia and RA remained unclear, but it was speculated that there was a common autoimmunologic factor between preeclampsia and RA [49]. In addition, RA women had an elevated risk of spontaneous abortion. Evidence has shown the higher rates of abortion after RA diagnosis [26, 55], especially in RA women with high disease activity during pregnancy [9].

Our analysis showed that RA was associated with the odds of SGA and LBW in infants. Strouse et al. have found that the odds of SGA were significantly increased in women with RA [3]. Some hypotheses explained how high disease activity led to LBW, such as high maternal cortisol level, vasculopathy, and high inflammatory cytokines levels that downregulate the activity of placental 11-hydroxysteroid dehydrogenase type 2 [56]. Moreover, we found that infants born from RA mother had a higher risk of congenital anomalies, and the heterogeneity of the pooled result was low (42.3%). Huang et al. also found the significant association between RA and congenital anomalies, and the heterogeneity of the pooled result was higher (78.4%) [8]. The reason for the difference in the heterogeneity for congenital anomalies may be that more studies included in our meta-analysis for this outcome, and the sample size was bigger, which might improve the statistical power. One study reported that children of a parent with RA had two-fold increased odds of type 1 diabetes [57]. We reported the similar finding that infants born from RA mother displayed the higher risk of type 1 diabetes. Asthma was a chronic inflammatory disease of the pulmonary system, and fetal allergic immune system was related to maternal inflammations [58]. Therefore, the status of maternal immune system was linked to childhood asthma. Yang et al. reported an elevated chance of asthma in children born to mothers with maternal RA [41], which was consistent with our analysis.

A significantly increased risk of cesarean delivery was found in women with RA in this meta-analysis. Huang et al. have reported that RA increased the risk of cesarean delivery, and speculated that differences in cesarean delivery rare may be associated with the disease activity [8]. A study by Zbinden et al. reported a significant association between high disease activity and the high odds of caesarean delivery in women with RA, implying that inadequate control of disease activity may lead to caesarean delivery [42]. In our meta-analysis, through quantitative analysis, we found that high disease activity of RA was associated with the higher risk of cesarean delivery. Our meta-analysis further provided the evidence for the association between active RA and cesarean delivery. We also found that the risk of premature delivery was increased with the higher disease activity of RA. Al Rayes et al. have reported the significant association between high disease activity and premature delivery [9]. The similar result was found in the study of Smith et al. [10]. Our findings suggested that a better disease control may be beneficial to improve the pregnancy outcomes of women with RA.

Our systematic review and meta-analysis further compare the pregnancy outcomes in women with RA and without RA, and also quantitively analyzes the association between disease activity and the risk of pregnancy outcomes in women with RA. There are some limitations in this study. First, heterogeneity may be caused by the different ages, onset time, and disease duration in included population, while data reported in the included studies are not enough to support us to conduct subgroup analysis for further exploration. Second, maternal smoking and drinking habits, family history, and rheumatoid treatment during pregnancy also affect maternal and infant outcomes. Since above information is not stated in all original studies, these data cannot be included in this meta-analysis. Third, disease activity is assessed using DAS28, HAQ-DI, PS, and PGS. Due to the limited number of studies, DAS28 and HAQ-DI are used for quantitative analysis. In the future, more studies are needed to further verify our findings.

## Conclusion

Our meta-analysis showed that high disease activity of RA was associated with the increased risk of adverse pregnancy outcomes. RA women preparing for pregnancy should pay more attention to control the disease activity. It is essential to strengthen communications between patients, obstetricians, and rheumatologists to develop individualized treatment plans for women with high disease activity of RA who are preparing for pregnancy.

## Data Availability

The datasets used and/or analyzed during the current study are available from the corresponding author on reasonable request.

## Abbreviations

RA: Rheumatoid arthritis
LBW: Low birth weight
NOS: Newcastle-Ottawa Scale
JBI: Joanna Briggs Institute
OR: Odds ratio
WMD: Weighted mean difference
